# Is Sham Training Still Training? An Alternative Control Group for Attentional Bias Modification

**DOI:** 10.3389/fpsyg.2020.583518

**Published:** 2020-11-04

**Authors:** Marika Tiggemann, Eva Kemps

**Affiliations:** School of Psychology, Flinders University, Adelaide, SA, Australia

**Keywords:** control group, appetitive stimuli, modification, attentional bias, sham training

The tendency to selectively attend to environmental stimuli congruent with self-relevant concerns has been documented across a wide range of mental and physical health domains. In particular, such attentional biases have now been demonstrated for a number of appetitive and/or addictive substances, including cigarettes among smokers (e.g., Waters et al., 2003), alcohol in heavy drinkers (e.g., Townshend and Duka, 2001) and high-calorie food in obese individuals (e.g., Kemps et al., 2014). The most common way to demonstrate attentional bias is implicitly, via the dot probe task (Posner et al., 1980), in one version of which pairs of words or pictures are presented briefly, followed by a small dot in the spatial location of one of the stimuli. The participant's task is simply to determine the location of the dot probe as quickly as possible. When the pairs consist of one self-relevant stimulus (e.g., a picture of beer) and one neutral stimulus (e.g., a picture of a glass of water), attentional bias is demonstrated by speeded detection of probes replacing the self-relevant stimulus relative to the neutral stimulus.

More recently, research has extended the protocol initially used to successfully modify attentional bias for threat-related stimuli in anxiety (MacLeod et al., 2002) to addictive and craved substances. In this modified dot probe task, a contingency is introduced whereby the dot probe appears disproportionately (90–100%) in place of the neutral word or picture, thereby training attention away from the substance-relevant cue. The central idea is that over time the repeated practice of responding to probes in the spatial location of the neutral cue induces a shift of attention (as indicated by relative response latencies) away from the substance-relevant cue and toward the neutral cue. This is seen as an implicit and gradual process (MacLeod et al., 2002; Koster et al., 2009; Kemps et al., 2014), although there is still considerable uncertainty as to the precise mechanisms mediating the effect (Heeren et al., 2013). A number of reviews and meta-analyses have now shown some effectiveness for attentional bias modification in appetitive domains, but effects are small and conclusions limited by methodological weaknesses (Beard et al., 2012; Turton et al., 2016; Jones et al., 2018; Boffo et al., 2019). In addition, some reviews have questioned the clinical utility of attentional bias modification, concluding that there is insufficient evidence that positive effects on attentional bias translate into any effect on addiction outcomes (Christiansen et al., 2015; Cristea et al., 2016).

One identified limitation lies in the use of different and suboptimal control conditions (Turton et al., 2016; Jones et al., 2018; Boffo et al., 2019). Following MacLeod et al.'s protocol, some studies have implemented comparison conditions where the contingency is reversed, i.e., the dot probe disproportionately replaces the substance-relevant stimulus (training attention toward the substance-relevant cue). Such a protocol both maximizes observed differences between “attend” and “avoid” conditions and means that they cannot be attributed unambiguously to a reduction in attentional bias in the “avoid” group. In addition, an “attend” condition is often not viable in studies of addictive substances for ethical reasons. Other studies have used no training (treatment as usual), wait-list, or unrelated tasks as control conditions. Over time, however, researchers have settled on what has come to be called “sham training” as the optimal control. In sham training, the dot probe replaces the substance-relevant stimuli and neutral stimuli with equal frequency (50/50), meaning that attention is not particularly directed to either. Sham training has become the “gold standard” control condition because it is so well-matched to the attentional re-training experimental condition in both stimulus exposure and response requirements; only the contingency is different. Perhaps not surprisingly, fewer significant effects of attentional bias modification are observed when contrasted against the more stringent sham training than against other control conditions (Beard et al., 2012).

While widely accepted as the best available control condition, there have recently been some questions raised about the nature of sham training. In particular, a pattern noted across multiple domains is that the lack of significant difference between experimental and control groups often comes about because both groups improve (Cristea et al., 2015). This seems especially the case with clinical samples (Blackwell et al., 2017). Interestingly, a similar pattern has been noted in studies of approach bias modification (Kakoschke et al., 2018). This observation has led some researchers to suggest that sham training may in fact have an active component, rather than offering, as assumed, an “inert,” “neutral,” or “placebo” training, that should not of itself change cognitive biases in any specific direction. Cristea et al. (2015) suggest the operation of various demand characteristics, while Boffo et al. (2019) suggest a more general exposure or desensitization process, whereby continued exposure to substance-related stimuli (irrespective of any contingency or response) may result in participants becoming less sensitive over time to the motivational meaning of the substance-related cue.

When used as a control condition, sham training is typically characterized as “no contingency” training. However, in the dot probe (and other tasks), contingency is a continuous variable, running from 100/0 (dot replaces neutral stimulus 100% of the time) to 0/100 (dot replaces substance-relevant stimulus 100% of the time). Thus, when probes replace the neutral and substance-related stimuli with equal frequency, this is better described as a 50/50 (rather than “no”) contingency. Accordingly, a few authors have suggested that sham training actively trains equal attention to substance-relevant and neutral stimuli and thereby may affect control over attention for substance-related stimuli (e.g., Schoenmakers et al., 2010; Badura-Brack et al., 2015; Khanna et al., 2016). Others have suggested that sham training serves to train participants to ignore emotional stimuli when confronted with them (Gladwin, 2017; Gladwin et al., 2019). In line with these ideas, the sham training protocol has sometimes been reconceptualized and renamed as “attentional control training,” and viewed as a more top-down goal-directed process (Gladwin, 2017). In combat veterans, such attentional control training has been shown to be more effective for PTSD symptom reduction than traditional attentional bias modification (Badura-Brack et al., 2015; Khanna et al., 2016). These latter authors view attentional control training as particularly effective at normalizing attention allocation, although the exact cognitive mechanism(s) underlying the effect of exposure to a 50/50 contingency (conceptualized either as sham training or attentional control training) is yet to be clarified.

To the extent that exposure to the 50/50 contingency can be seen as balancing attentional allocation between relevant and neutral stimuli (Badura-Brack et al., 2015), the size of effect of sham training will logically depend upon an individual's initial level of attentional bias for the target stimulus category, be it alcohol, cigarettes, or food. To elaborate, for people who have no initial attentional bias (approximately equal attention to substance-relevant and neutral stimuli), sham training should produce little change. But for individuals with a strong initial attentional bias toward a particular substance, the gradual training of equal attention to substance-relevant and neutral stimuli involved in sham training represents a substantial shift in relative attention away from the substance-relevant stimuli (where the majority of their initial attention was directed). Thus, sham training with its 50/50 contingency will serve to decrease attentional bias for these individuals. This account is able to explain the observation that sham training tends to lead to greater improvements in clinical samples (Blackwell et al., 2017). This trend, in turn, accounts for the general conclusion that attentional bias modification is more successful (when sham training is the control) in unselected or analog samples than it is in clinical samples (Cristea et al., 2015).

Of course, it is difficult to conceptualize a better alternative control task than sham training. Schoenmakers et al. (2010) developed a novel categorization task that avoided the 50/50 contingency. Ideally, what is required is a neutral control protocol that does not manipulate contingencies between stimulus categories and responses (a truly “no contingency” training condition), but otherwise matches the stimulus exposure and response requirements of attentional bias retraining. We have attempted to devise such a protocol and put it forward here for scrutiny. In our version of sham training, which we call sham-n training (“n” for “neutral,” or “no contingency”), instead of stimulus pairs consisting of one substance-relevant and one neutral picture, they are constructed to be of either two substance-relevant pictures or two neutral pictures. On half the trials, participants are presented with two substance-relevant pictures, and on the other half of trials with two neutral pictures (in random order and appropriately counter-balanced). As before, the probe is set to replace the left and right pictures with equal frequency (50/50) and participants need to determine the location of the probe. Thus, sham-n training directs attention equally to stimuli within a category, but not across categories. In a nutshell, participants receive the same number of trials and amount of stimulus exposure and are required to make the same judgements (probe location) and associated motor responses in sham-n training as in the original task. But in sham-n training, there is absolutely no relation between stimulus category and response.

The critical test of the sham-n training protocol is that, as befits an inert control condition, it should not lead to any change in attentional bias. In our first trial with an undergraduate sample, we found that this control condition resulted in no change in attentional bias for chocolate. The mean of 6.64 after sham-n training was very similar to the mean of 6.61 before sham-n training. This contrasted with results for the “avoid chocolate” group who experienced a significant reduction in attentional bias for chocolate (and “attend chocolate” group who experienced a significant increase). We also have preliminary data from a small field study of people trying to lose weight, where sham-n training (administered multiple times via smartphone app) produced no significant change in attentional bias for unhealthy foods, whereas the experimental training did. Although these results would carry more weight if they were contrasted with traditional sham training or some other control condition, they can be taken as preliminary proof-of-concept for our task. An important next step would be to test the protocol with individuals who demonstrate elevated levels of attentional bias toward any substance, such as clinical samples.

In sum, we present sham-n training as a potential control protocol in studies of attentional bias modification. The same logic could potentially be applied to developing control protocols for the modification of other cognitive biases (e.g., approach and interpretation biases). As pointed out by Blackwell et al. (2017), control conditions rarely generate the interest or excitement of active training conditions but are nevertheless critically important to the interpretation and value of their results. We welcome feedback on the sham-n training protocol and, of course, we welcome other investigators trialing it in their own settings.

## Author Contributions

MT and EK conceptualized all aspects of the opinion piece together. All authors contributed to the article and approved the submitted version.

## Conflict of Interest

The authors declare that the research was conducted in the absence of any commercial or financial relationships that could be construed as a potential conflict of interest.
